# Confidentiality in medical images through a genetic-based steganography algorithm in artificial intelligence

**DOI:** 10.3389/frobt.2022.1031299

**Published:** 2022-10-06

**Authors:** Eduardo Vazquez, Stephanie Torres, Giovanny Sanchez, Juan-Gerardo Avalos, Marco Abarca, Thania Frias, Emmanuel Juarez, Carlos Trejo, Derlis Hernandez

**Affiliations:** ^1^ Instituto Politécnico Nacional ESIME Culhuacan, Coyoacan, Mexico; ^2^ Tecnológico Nacional de México, Tecnológico de Estudios Superiores de Ecatepec, Estado de México, Mexico

**Keywords:** genetic algorithms, steganography, medical images, confidentiality, artificial intelligence

## Abstract

Nowadays, image steganography has an important role in hiding information in advanced applications, such as medical image communication, confidential communication and secret data storing, protection of data alteration, access control system for digital content distribution and media database systems. In these applications, one of the most important aspects is to hide information in a cover image whithout suffering any alteration. Currently, all existing approaches used to hide a secret message in a cover image produce some level of distortion in this image. Although these levels of distortion present acceptable PSNR values, this causes minimal visual degradation that can be detected by steganalysis techniques. In this work, we propose a steganographic method based on a genetic algorithm to improve the PSNR level reduction. To achieve this aim, the proposed algorithm requires a private key composed of two values. The first value serves as a seed to generate the random values required on the genetic algorithm, and the second value represents the sequence of bit locations of the secret medical image within the cover image. At least the seed must be shared by a secure communication channel. The results demonstrate that the proposed method exhibits higher capacity in terms of PNSR level compared with existing works.

## 1 Introduction

Currently, the growing use of Internet of Things (IoT) and Internet of People (IoP) technologies has meant that information between users has to be increasingly safer, for example: in video surveillance, vehicle location, medical diagnoses through images, etc. Therefore, sensitive data is concerned with user privacy and is more vulnerable to disclosure and tracking. For this reason, security and privacy are of great importance (Hernández et al., 2019) and there are great challenges for the preservation of information security. In particular, the processing of medical images generates a large amount of information, much of which must be kept confidential. To achieve this goal, several authors have used steganographic techniques to hide sensitive information in images. Image steganography techniques can be divided into two groups according to the manner in which the secret message is inserted. On one hand, there is steganography in the spatial domain where the secret message is inserted directly into the pixels of the image, within this category is the least significant bit technique (Chan and Cheng, 2004). On the other hand, there is steganography in the frequency domain. Within this category are the fast Fourier transform and the discrete cosine transform. These algorithms are used to transform image data to their coefficients in the frequency domain and perform information hiding (Rekik et al., 2012). Although steganography in the spatial domain provides greater insertion capacity than steganography in the frequency domain, the latter technique provides greater robustness against malicious processing of the stego-image. To date, several authors have used steganography in the spatial domain. For example, Chandrasekaran, Arumugam, and Rajkumar (Chandrasekaran et al., 2015) propose a method for optimal pixel selection in image steganography based on a genetic algorithm, to minimize the visual perception of image degradation as much as possible. The method proposed by Chandrasekaran et al. uses the random selection of pixels to insert the message to hide through logistic mapping. Subsequently, the selection of the pixels is optimized by a genetic algorithm to minimize the distortion of the stego-image, the fitness function used in the proposed method is the PSNR (Peak Signal-to-Noise Ratio). On the other hand, Ghasemi and Shanbehzadeh (Ghasemi and Shanbehzadeh, 2010) propose a data hiding scheme in 8 × 8 bit blocks using a cover image. The proposed scheme uses a mapping function based on a genetic algorithm and OPAP (Optimal Pixel Adjustment Process) (Chan and Cheng, 2004) in order to reduce the margin of error between the stego-image and the carrier image. Janabi and Al Shourbaji (Al-Janabi and AlShourbaji, 2016), propose a method to hide multiple images within a cover image, making their proposal novel by employing a genetic algorithm to generate the stego-key *K*, which is represented by a matrix called “mixing matrix”. Mstafa and Elleithy propose a secure video steganography scheme using Hamming Code (Mstafa and Elleithy, 2014), where nine decompressed video sequences are used as the cover of the secret message and one image (logo) is used as the hidden message. In the proposal by Mstafa et al. the pixel positions of the cover and the secret message are randomly reordered using a private key which increases the security of their system. Swathi and Jilani in (Swathi and Jilani, 2012) propose a video steganography scheme using the LSB substitution method based on the private key steganography protocol using polynomial equations with different coefficients as the private key. Analyzing the previous works, it can be observed that the cover image undergoes measurable modifications through the PSNR level. Therefore, there are currently great challenges to hide messages within an image without modifying it. For this reason, in this work a steganography scheme is presented, which provides a high capacity for inserting a medical image into another cover image, guaranteeing that the stego-image is not altered. The proposed scheme uses a genetic algorithm for the selection of pixels that will host the secret medical image. The main contribution of our image steganography scheme is to host a high number of bits in the spatial domain of the medical image, without the need to alter it, optimally fulfilling the requirement of imperceptibility in images.

## 2 Genetic algorithms

The genetic algorithm (GA) developed by Holland (Holland, 1962) is a metahuristic inspired by Neo-Darwinism that simulates the biological process of adaptation of living beings to their environment for problem solving.

**FIGURE 1:**
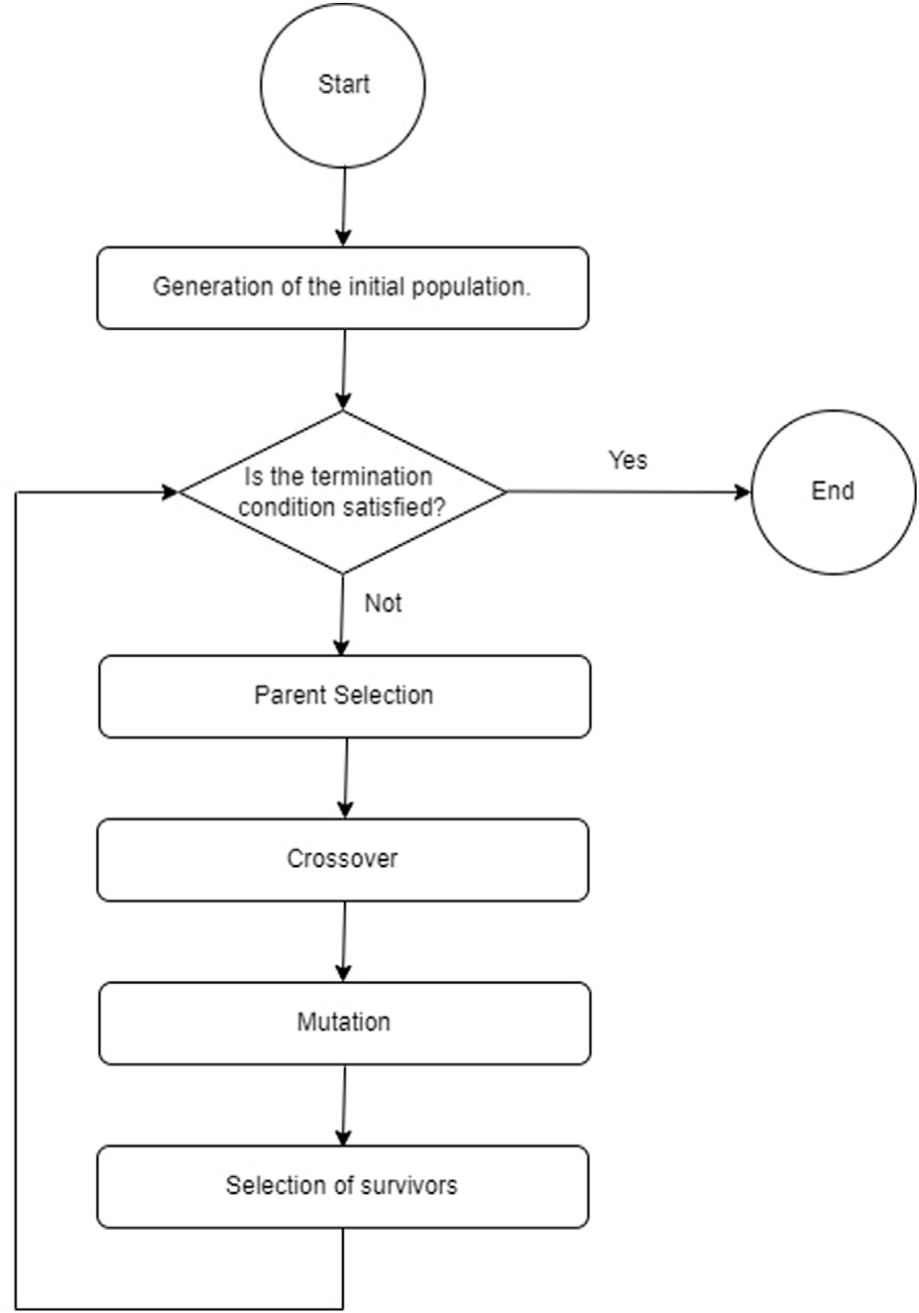
General scheme of the genetic algorithm.

The genetic algorithm basically consists of generating a random population of candidate solutions to the problem in question, to be subjected to the genetic crossover and mutation operators for a certain number of generations. At the end of the evolutionary process, the best adapted solutions are the ones that will prevail. For this reason, GAs are used in a significant way to carry out optimization processes. The mechanism represented in Figure 1 is executed cyclically for a given number of generations, or until the termination condition of the genetic algorithm is met.

The main components of the genetic algorithm are:

• Representation: The goal of the representation is to map individuals from a real-world perspective to a representation that the computer is capable of manipulating. Phenotype is defined as the set of parameters that represent a chromosome, so that each phenotype constitutes a possible solution to a given problem, while its coding (the individuals within the evolutionary algorithm) are called genotypes.

The inverse mapping of genotypes and phenotypes is known as decoding, therefore it is important to use a representation that has the ability to be invertible.

**FIGURE 2:**
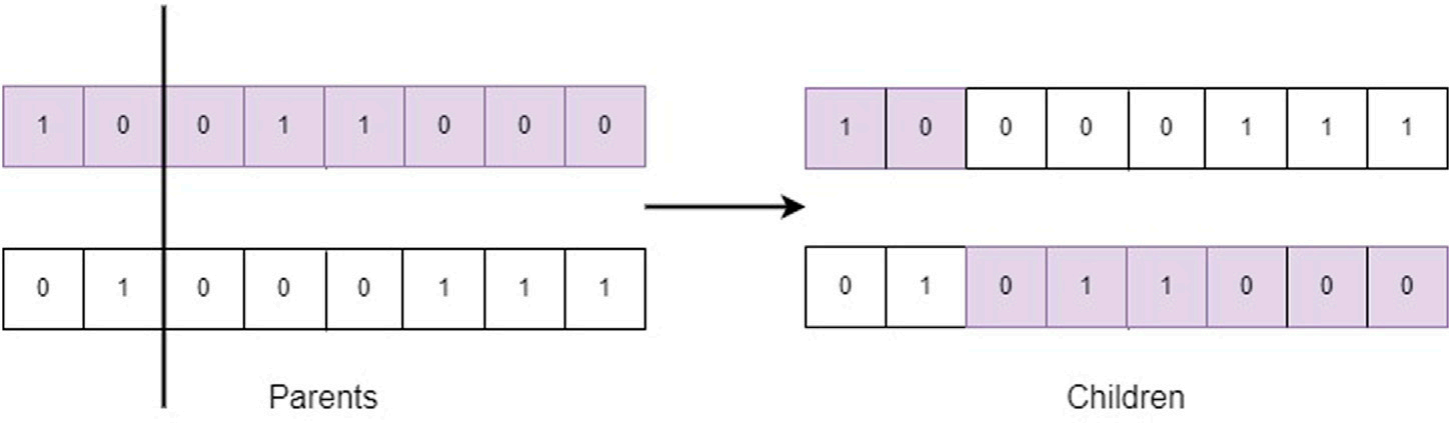
One-point crossover.

• Population: It contains the possible solutions to the problem to be solved and forms the set of individuals responsible for carrying out the evolutionary process. In the design of the genetic algorithm, the population size must be defined, which will remain constant throughout the evolutionary algorithm.
• Fitness function: The fitness function evaluates the quality of the genotypes according to their degree of adaptation with respect to the problem to be solved. This function is defined in the phenotype space, therefore, its decoding is necessary to be able to evaluate it.
• Parent Selection: The parents that will be submitted to the GA crossing operator are selected probabilistically. The objective is to select the best adapted individuals based on the quality of their fitness in order to increase the fitness of individuals in the next generation, although always there should be the possibility of choosing individuals with a low fitness value to avoid the problem of premature convergence in the GA.
• Crossing: The genetic crossing operator emulates the biological process of reproduction, where the exchange of genetic information of a pair of individuals (parents) is carried out to generate two children. Figure 2 represents the single point crossover mechanism.

Generally, the crossover rate *P*_*c*_ ∈ [0.6, 0.9] (Whitley, 1994).

**FIGURE 3:**
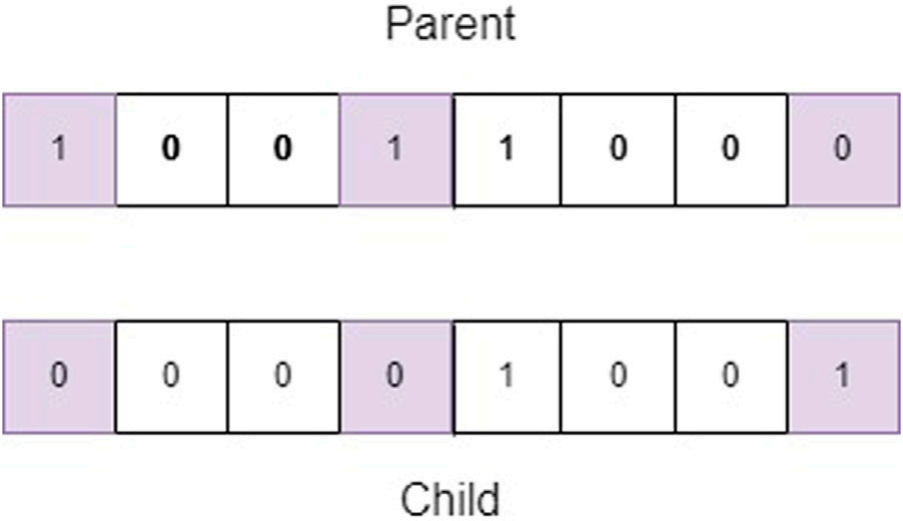
Uniform mutation.

• Mutation: The genetic mutation operator acts directly on the genotype and is stochastic in nature, since the resulting children depend on random variations. To apply the mutation operator, a low mutation rate *P*_*m*_ (Eiben and Smith, 2015) is generally used. In (Davis, 1991) it is recommended to use a mutation rate *P*_*m*_ in the range [0.01, 0.001]. An example of mutation is shown in Figure 3, in it the bits of positions 0, three and seven are mutated. In the upper part, the chromosome before being mutated is shown, and in the lower part, the result of the mutation.
• Selection mechanism: The purpose of survivor selection is to choose the fittest individuals who will be passed on to the next generation in the evolutionary algorithm.

## 3 Steganography

The term steganography refers to covert writing by its greek words “steganos” meaning covered and “graphos” meaning writing. However, steganography has also been defined as the art and science of covert communication (Provos and Honeyman, 2003); Covert communication is achieved by hiding information within some carrier object, nowadays it is common to use digital media as cover objects.

All digital files can be used for steganography but it is recommended to use objects with a high degree of redundancy in order to alter the redundant bits without such alteration being easily detected. The digital files that mainly meet the characteristic of having a high level of redundancy are digital images and audio, in the literature it is commonly mentioned that the digital media that can be used in steganography are: text, images, audio/video and network protocols.

### 3.1 Steganography system

**FIGURE 4:**
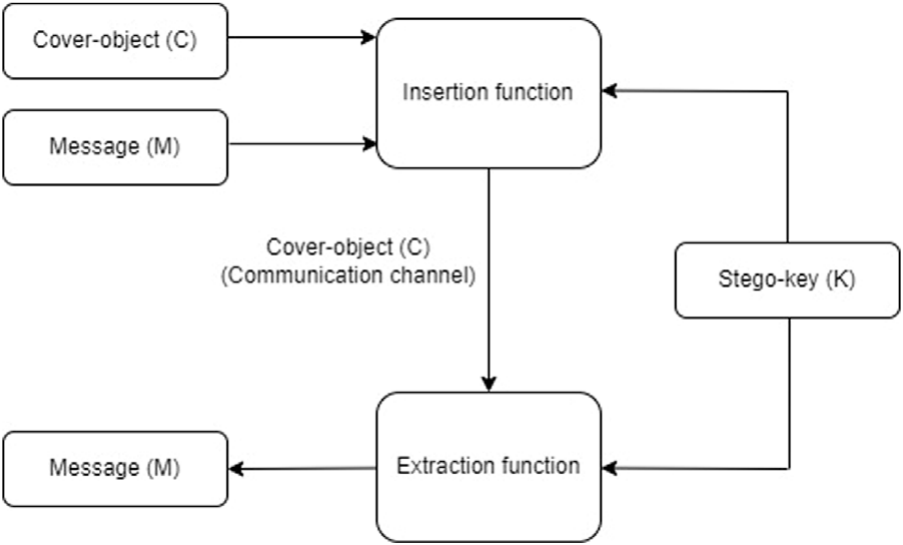
Steganography system.

A steganography system is the set of elements that allow covert communication to be carried out (See Figure 4). The basic terminology of steganography is listed below:

• Message (*M*): The secret message to hide inside a cover object.
• Stego-key (*K*): It is additional secret information that may be necessary in the process of hiding and extracting a message.
• Cover-object (*C*): It is the cover object that will hide the secret message.
• Stego-object (*C*′): It is the output of the insert function of the message to hide, the stego-object contains the secret message hidden in the cover-object.
• Insertion Algorithm: Refers to the process of hiding the secret message into the cover-object, by means of an insertion function.
• Extraction algorithm: It is the process of extracting the secret message from the cover-object, by means of an extraction function.

### 3.2 Steganography protocols

In the literature there are three classes of steganography protocols: pure steganography, private key steganography and public key steganography, each of which will be described below.

#### 3.2.1 Pure steganography

In this case, the prior exchange of information is not necessary to be able to specify the insertion or extraction of a secret message; that is, the exchange of a stego-key is not required in this type of steganography protocol, but it is necessary that both sender and receiver have exclusive knowledge of the secret message insertion and extraction algorithms.

Formally, pure steganography is defined by *S* =< *C*, *M*, *D*, *E* > (Katzenbeisser and Petitcolas, 2000), where *C* is the set of possible cover-objects, *M* is the set of secret messages with |*C*|≥|*M*|. The secret message insert function is given by *E*: *C* × *M* → *C*′ and the message extract function is *D*: *C*′ → *M*, with the property that *D* (*E* (*c*, *m*)) = *m*, for all *m* ∈ *M* and *c* ∈ *C*.

#### 3.2.2 Private key steganography

This protocol requires the existence of a private stego-key between the receiver and the sender. It is similar to symmetric cryptography, where the sender inserts a secret message using the secret key, and if the receiving party has this key, then the secret message can be extracted.

The formal definition of private key steganography (Katzenbeisser and Petitcolas, 2000) is as follows: Let *S* =< *C*, *M*, *K*, *D*_*k*_, *E*_*k*_ >, where *C* is the set of possible cover-objects, *M* is the set of secret messages with |*C*|≥|*M*| and *K* is the set of secret keys. The secret message insert function is *E*_*k*_: *C* × *M* × *K* → *C*′ and the message extract function is *D*_*k*_: *C*′ × *K* → *M*, with the property that *D*_*k*_ (*E*_*k*_ (*c*, *m*, *k*), *k*) = *m*, for all *m* ∈ *M*, *c* ∈ *C* and *k* ∈ *K*.

#### 3.2.3 Public key steganography

The public key steganography protocol is similar to public key cryptography in that it requires the use of two keys: one public and one private. In the context of steganography, both the sender and the receiver must have both keys.

## 4 Genetic-based steganography algorithm

**FIGURE 5:**
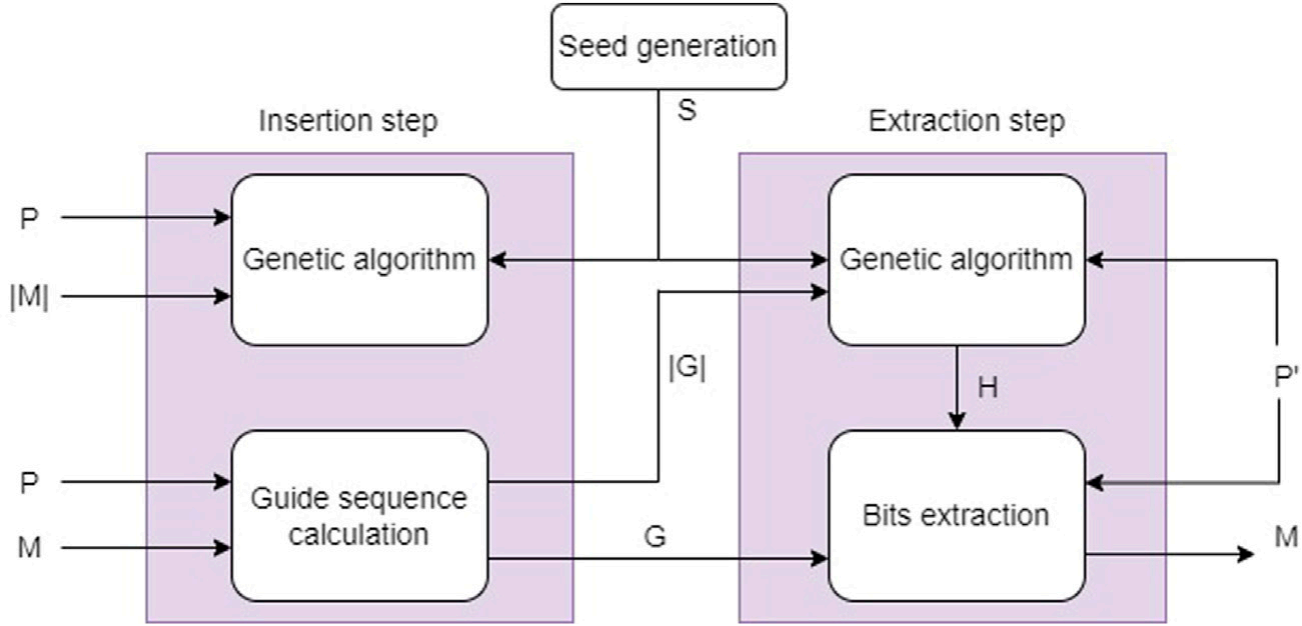
Genetic-based steganography method.

In Figure 5 the proposed steganographic system is shown. This method allows us to hide the original image *I* in a cover image *P* in any of the following formats: GIF, JPEG, and BMP. This system is based on the private key steganography protocol (Katzenbeisser and Petitcolas, 2000), so the sender is required to share the same key with the receiver in order to carry out the search and extraction of the secret image *I*. In our proposal, the key *K* = (*S*, *G*) is composed of two parts: *S* denotes an integer value that is used to generate random values in the genetic algorithm. On the other hand, *G* represents the sequence of the positions of the bits that contain the image *I*. We have called *G* the guide sequence.

Two steps are performed in the search phase. First, a genetic algorithm is used to determine the location of the pixels in the cover image *P* that will contain the secret image *I*. This GA generates its random values from the seed S. We have named this sequence of pixels host pixels *H*. As a second step, the guide sequence *G* is calculated with Algorithm 2 (see Section 4.2). The extraction phase also contemplates two steps. Initially, the host pixels *H* are computed with the same genetic algorithm used in the seach stage. This GA used the seed *S*, the stego-image *P*′ and the size of the guide sequence |*G*| (data required to create chromosomes of this size in the GA). Finally, the bits that allow the secret image *I* to be recovered from the guide sequence *G* and the set *H* are extracted.

It is important to mention that the cover image *P* and the stego-image *P*′ constitute exactly the same image, since our method does not need to alter the original image to hide the image *I* in *P*. In fact, this is the main contribution of our work.

### 4.1 Genetic algorithm for host pixels selection

In our method, the genetic algorithm is used to carry out the selection of the pixels that will host the secret image *I*. There is a one-to-one correspondence between the bits that make up the secret image *I* and the host pixels; that is, for each bit in *I*, a host pixel must be chosen to contain it.

Through the GA developed in this proposal, |*I*| pixels of the cover image are probabilistically selected to host |*I*| bits of the secret image *I* (|*I*| denotes the number of bits of the image *I*). The sequence of locations of the selected pixels make up the chromosome of the individual *i* in the genetic algorithm. Therefore, each gene on the chromosome of a given individual can be mapped to a pixel of the cover image *P*. We denote by *C*_*i*_ the chromosome of the individual *i*, in such a way that *C*_*i*_ = *V*_*i*,*j*_, where:

• *i* represents an individual (0 ≤ *i* < *N*).
• *j* represents the gene position (0 ≤ *j* < |*I*|).
• *V*_*i*,*j*_ ∈ [0, *wh* − 1] denotes the *jth* gene of the *ith* individual, where *w* and *h* represent the width and height of the cover image, respectively.

**FIGURE 6:**
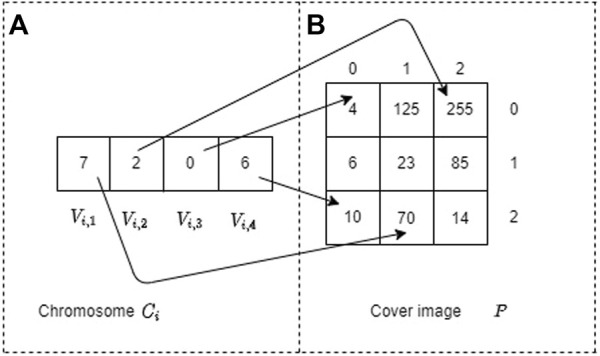
Mapping example of the chromosome *C*_*i*_ to the cover image *P* of 3 × 3. Part **(A)** represents the chromosome *C*_*i*_ and part **(B)** denotes the cover image *P*.

Therefore, the row and column of the image *P* are defined by $\left\lfloor \frac{V_{i,j}}{w}\right\rfloor$ and (*V*_*i*,*j*_ mod  *w*), respectively (mod denotes the modulus or rest of the division). Figure 6 shows a mapping example of the values of the chromosome *C*_*i*_ to the cover image *P* of 3 × 3 pixels. For example, if we have that the gene *V*_*i*,1_ = 7, then the row and column in the image *P* are determined by $\left\lfloor \frac{V_{i,1}}{w}\right\rfloor =\left\lfloor \frac{7}{3}\right\rfloor =2$ y (*V*_*i*,1_ mod  3) = (7 mod  3) = 1, respectively.

It is worth mentioning that the size of a chromosome must equal the size of the secret information because each bit of the secret message must correspond to the position of a gene within the chromosome.

#### 4.1.1 Characteristics of the proposed genetic algorithm

In Algorithm 1 the proposed genetic algorithm is shown. After iterating this algorithm for *GEN* generations, an individual from the last generation is randomly selected with a uniform distribution, which corresponds to the host pixels sequence *H*. In this procedure, the single-point crossover is used, which was shown in Figure 2. The seed *S* is used to generate, with a uniform and random distribution, the crossing points in the range 0 < *x* < |*I*|.

**Require:** |*I*|: Bits number of the secret image *I*, *P*: Cover image, *S*: Seed used to generate random numbers.

**Ensure:** *H*: Host pixels sequence.

Algorithm 1 Genetic algorithm for host pixels selection.

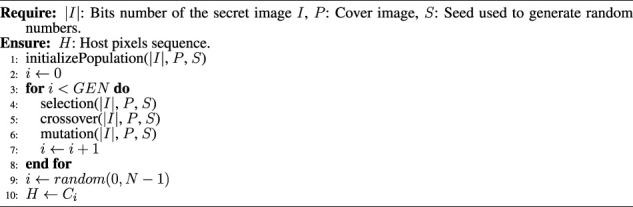

The mutation is carried out by applying the random resetting technique (Eiben and Smith, 2015). In this way, the chromosome resulting from the mutation for the individual *C*_*i*_ = *V*_*i*,*j*_ (0 ≤ *i* < *N* y 0 ≤ *j* < |*I*|) is given by $P_{i}^{\prime }=V_{i,j}^{\prime }$ , in such a way that:

$$V_{i,j}^{\prime }=\left\{\begin{array}{cc} rand\left(0,wh-1\right) & \text{if }m\leq P_{m}\\ V_{i,j} & \text{ otherwise} \end{array}\right.$$

Table 1 shows the characteristics of the proposed genetic algorithm. In this GA an integer representation is used, with generational population management (Eiben and Smith, 2015), one-point crossover, mutation based on random resetting technique and tournament selection mechanism. Because the only goal of the genetic algorithm in our proposal is to establish a random walk that defines the sequence of host pixels H, there is no problem in using other selection methods, such as stochastic and tournament selection. Consequently, there is no problem in using other crossover or mutation methods in the GA.

**TABLE 1:** Characteristics of the proposed genetic algorith.

GA component	Technique used
Representation	Integer
Population management	Generational
Crossover	One-point
Mutation	Random resetting
Selection mechanism	Tournament

The fitness function (FA) of the GA favors those individuals that successfully locate all the host pixels to store all the bits of the secret image. For each bit found favorably in the host pixel, the fitness function increases its value by one. If the FA does not find the desired bit in the host pixel proposed by the GA, then it does not increment its value by one. There are two possible scenarios; for example, if the GA is looking for the zero bit in the host pixel that stores the value 255 or if it is looking for the one bit in the host pixel that corresponds to the value zero. Therefore, the fitness function has a probability of 254/256 of finding a given bit in a pixel of the cover image. The objective function is given by:

$$fitness=\overset{\left|G\right|}{\underset{i=0}{\sum }}1$$

Where the element *i* (which corresponds to gene *i* of an individual’s chromosome) is only considered if the value of the associated pixel has a value in the range [1, 255]. *G* denotes the guide sequence.

### 4.2 Generation of the guide sequence

Each bit of the secret image *I* corresponds to a location within a given host pixel of the carrier image *P*. We have called the sequence of all these locations the **guide sequence**. Algorithm 2 shows the method for calculating this guide sequence. In lines 1 − 15 the algorithm loops through all the bits that make up the image *I*. In line 2, the value of *C*_*i*,*j*_ is obtained in binary notation and stored in the string *b*. Let us recall that the *C*_*i*,*j*_ corresponds to the gene *V*_*i*,*j*_, where 0 ≤ *i* < *N* and 0 ≤ *j* < |*I*| (See 6). The idea of lines 5 through 11 is to loop through all the bits of *C*_*i*,*j*_ to find the *jth* bit of *I* on line 6. If this bit is found, the value of the guide sequence *G*_*i*,*j*_ is equal to the index *z* at which the *jth* bit of *I* was found. If the bit *j* is not found, then the condition that *G*_*i*,*j*_ = 7 is established, so when the extraction of the secret image is carried out, the requested bit will correspond to the inverse of the eighth bit stored in *G*_*i*,*j*_.

**Require:** *I*: Secret image, *H*: Guide secuence (*C_i_*), *P*: Cover image.

**Ensure:** *G_i,j_*: Guide sequence vector of the individual *i*

Algorithm 2 Calculation of the guide sequence *G*_*i*,*j*_.

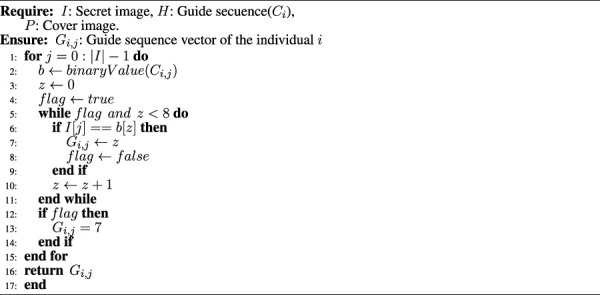

### 4.3 Proposed search algorithm

The steps involved in the proposed search algorithm are shown below.

1) Choose the cover image *P* of size *wh* pixels, where *w* and *h* are the width and height of the image, respectively.
2) Convert the secret image *I* to binary representation.
3) Sender and receiver should agree the seed *S* to generate random numbers.
4) Apply genetic Algorithm 1 to find the host pixel sequence *H* corresponding to the pixel chromosome *C*_*i*_, where *i* is a random value from 0 to *N* − 1. In the case of RGB type images (JPEG images, for example), one of the three channels is randomly selected to work on.
5) Apply Algorithm 2 to obtain the guide sequence *G*_*i*,*j*_ of the individual *i* chosen in the previous point (0 ≤ *j* < |*I*| − 1).

### 4.4 Proposed extraction algorithm

The steps involved in the proposed extraction algorithm are shown below.

1) Use the same cover image *P* of size *wh* pixels that was used in the search algorithm.
2) Use the same seed *S* that was used in the search phase to generate random numbers.
3) Apply genetic Algorithm 1 to find the sequence of host pixels H corresponding to the chromosome *C*_*i*_, where *i* is a random value from 0 to *N* − 1. In the case of RGB type images (JPEG images, for example), one of the three channels is randomly selected to work on.

So far the steps are identical to the search algorithm.

4) Extract the bits from the secret image *I* using the guide sequence *G*_*i*,*j*_ and the host pixel sequence of the individual selected in the previous point.
5) Transform the bits that make up *I* to the original image.

## 5 Tests performed

**FIGURE 7:**
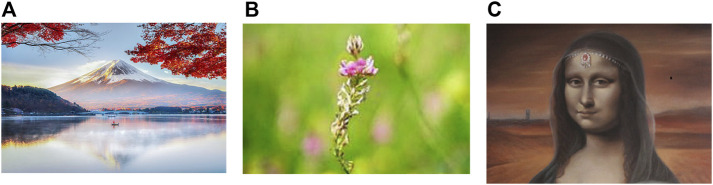
Figures **A–C** represents the set of cover images used in the tests performed.

In order to test the effectiveness of the proposed steganographic system, the cover images shown in Figure 7 were used. Table 2 shows the characteristics of these cover images and parameters of the genetic algorithm used in the tests performed.

**TABLE 2:** Cover image characteristics and parameters of the GA used in the tests performed.

Image	Dimensions	Formats used	Seed	GA parameters
Landscape	512 × 512	GIF, JPEG, BMP	10	*GEN* = 30, *P*_*m*_ = 0.30 *P*_*c*_ = 0.3 and *N* = 50
Flower	25 × 25	GIF, JPEG, BMP	500	*GEN* = 50, *P*_*m*_ = 0.40 *P*_*c*_ = 0.5 and *N* = 100
Mona Lisa	408 × 349	GIF, JPEG, BMP	3	*GEN* = 60, *P*_*m*_ = 0.35 *P*_*c*_ = 0.6 and *N* = 30

**FIGURE 8:**
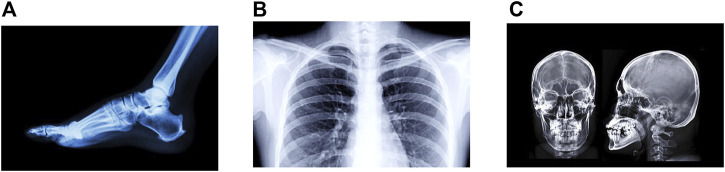
Figures **A–C** represents the secret images used in the experiments carried out.

30 medical images were used to test the proposed steganographic method. All of these images were successfully searched and retrieved into each of the cover images in Figure 7. Since the cover image does not suffer any alteration when hiding a secret image, in all cases the PSNR and SSIM measures were *∞* and 1, respectively, so the proposed steganographic system is not vulnerable against statistical attacks. Figure 8 shows three secret images used in the experiments performed.

### 5.1 Discussion of the results obtained

Various state-of-the-art methods have reported the measurements of MSE (Mean Square Error), PSNR (Peak Signal-to-Noise Ratio) and SSIM (Structural Similarity Index) to determine the level of distortion suffered by the original image when inserting a secret message. Caballero-Hernández et al. (Caballero Hernandez et al., 2019) report that 90% of state-of-the-art works use MSE and PSNR as quality measures, while SSIM is reported with a less significant percentage. For example, Naum et al. (Naoum et al., 2016) report values of MSE = 2.57 × 10^−5^ and PSNR = 105.68 dB, while Bhattacharyya et al. (Bhattacharyya et al., 2015) report SSIM value equal to 0.9332. However, in our method, the values obtained are excellent, since the PSNR and SSIM obtained were = *∞* and 1, respectively. Since there is no distortion in the stegoimage, the mean square error is 0.

Another aspect to take into account is the ability to insert data in the cover image. For example, Vaishali and Kajal (Vaishali and Kajal, 2015) had injection loads greater than 370, 000 bytes with a PSNR slightly above 50 dB. However, in our proposal, any number of bits can be searched into the cover image, since the function of the genetic algorithm is to find the host pixel that will contain each bit of the secret message, and the same pixel can store any number of bits (the limit is the size of the physical memory of the device being used).

Therefore, our method is innovative because it provides excellent PSNR, MSE, and SSIM values with a high insertion capacity. We also want to emphasize that the secret information is hidden within the guide sequence, although it is necessary to have the cover image and the host pixels sequence (determined by the genetic algorithm) in order to discover the secret message. The reason our method does not require altering the cover image P to hide the secret image I is because the probability of finding a given bit (zero or one) is 255/256. There are only two cases that it can fail, but these can be handled by adopting the convention that if the desired bit is not found, then it is the inverse of the bit in the seventh position of the host pixel, as can be seen in lines 12–14 of Algorithm 2.

It is worth mentioning that the purpose of the genetic algorithm proposed in this work is different from that of state-of-the-art works that also use GA. For example, Chandrasekaran et al. (Chandrasekaran et al., 2015) use the GA to locate those pixels that distort the cover image as little as possible, while our GA is only used to find a random sequence of pixels that will contain the secret image. In fact, in our method any random walk propose by the GA can be used to store the secret image without producing any distortion in the cover image.

### 5.2 Security analysis of the proposed method

Due to the fact that in our method the cover image does not undergo any alteration, the existing steganalysis techniques cannot be applied to this image to try to discover the secret message (Nissar and Mir, 2010). Therefore, we must analyze the security of our proposal in terms of the security of the private key. As mentioned above, the key *K* = (*S*, *G*) consists of two components: *S* denotes an integer value that serves as a seed to generate random values in the genetic algorithm, and *G* represents the guide sequence. Undoubtedly, *S* must be shared between sender and receiver through a secure communication channel (Menezes et al., 1996). If the guide sequence is also shared over a secure channel, then the security of our proposal is similar, for example, to that offered by symmetric cryptography (Menezes et al., 1996), in the sense that the confidentiality of the information is guaranteed as long as the adversary is unaware of the private key.

If the private key is not shared over a secure communication channel, the algorithmic complexity to find the secret message *M* is given by *O* (*wh* ^|*G*|^), where *w*, *h* and |*G*| denote the width and height of the cover image, and |*G*| represents the number of bits of the guide sequence *G*, respectively. The reason for this formula is that each value in the guide sequence can correspond to any of the *wh* possible locations within the cover image. This complexity belongs to the category of NP-Hard problems, whose main characteristic is that it contains decision problems that are at least as difficult as any NP problem (Menezes et al., 1996). Table 3 shows the algorithmic complexity of the private key for different sizes of the cover image and the guide sequence. In the first column, the dimensions of the image are shown; in the second column, the length of the guide sequence (in bits) is displayed. In the third column, the number of characters in the secret message (considering an eight-bit-per-character representation of the ASCII alphabet) corresponding to the size of |*G*|. In the fourth column, the maximum number of permutations that must be calculated to find out the secret message is shown. It can be seen that the algorithmic complexity to discover the secret information grows exponentially with the size of the guide sequence (and therefore of the secret message) and the size of the cover image *I*. It can also be seen that this problem is at least as hard as the traveling salesman problem, which is also NP-Hard, with complexity given by $O\left(\frac{1}{2}\left(n-\right)!\right)$ (Levitin, 2012).

**TABLE 3:** Algorithmic complexity of the proposed method.

Image dimensions	|*G*| (bits)	Number of characters	*O* (*wh* ^|*G*|^)
25 × 25	1,024	128	9.6 × 10^2,862^
25 × 25	2048	256	9.2 × 10^5,725^
512 × 512	1,024	128	3.8 × 10^5,548^
512 × 512	1,536	192	7.5 × 10^8,322^

## 6 Conclusion

The steganographic system proposed in this work allows us to hide medical images of arbitrary size in image files in GIF, JPEG, and BMP format, although our method can also be used to hide audio or text. This is achieved through a genetic algorithm that allows locating the host pixels that will contain the bits of the secret image. The proposal uses a private key composed of two values. The first is used as a seed to generate random values in the genetic algorithm; the second is composed of the positions within the host pixels that correspond to the values of the bits in the secret image. In our method, at least the seed must be shared over a secure communication channel. The main feature of this work is that the cover image is not modified during the image hiding process, so that our method is not vulnerable to statistical attacks. Future work contemplates investigating the capabilities of the present method to encrypt information in *n*-dimensional cover images generated through an evolutionary algorithm. It is also interesting to assign to the genetic algorithm the task of generating the cover image from the private key *S*. It would also be interesting to approach our method from the perspective of classification problems through neural networks, such as (Alfaro-Ponce and Chairez, 2020) and (Fuentes-Alvarez et al., 2022).

## Data Availability

The original contributions presented in the study are included in the article/Supplementary Material, further inquiries can be directed to the corresponding authors.
